# 1626. Group B Streptococcus detection rate and clindamycin resistance among reproductive-age women and early-onset sepsis in neonates in Korea from 2003 to 2022

**DOI:** 10.1093/ofid/ofad500.1461

**Published:** 2023-11-27

**Authors:** Ji-Hee Sung, Doo Ri Kim, Suk-Joo Choi, Soo-young Oh, Yae-Jean Kim, Cheong-Rae Roh, Areum Shin

**Affiliations:** Samsung medical center, Seoul, Seoul-t'ukpyolsi, Republic of Korea; Samsung medical center, Seoul, Seoul-t'ukpyolsi, Republic of Korea; Samsung medical center, Seoul, Seoul-t'ukpyolsi, Republic of Korea; Samsung medical center, Seoul, Seoul-t'ukpyolsi, Republic of Korea; Samsung Medical Center, Seoul, Korea, Seoul, Seoul-t'ukpyolsi, Republic of Korea; Samsung medical center, Seoul, Seoul-t'ukpyolsi, Republic of Korea; Samsung medical center, Seoul, Seoul-t'ukpyolsi, Republic of Korea

## Abstract

**Background:**

Group B Streptococcus (GBS) is the leading cause of early-onset sepsis (EOS) in neonates and intrapartum penicillin prophylaxis is recommended for pregnant women with GBS colonization to prevent vertical transmission. For pregnant women with penicillin allergy, clindamycin is recommended. In this study, we retrospectively examined the GBS detection rate and clindamycin resistance rate among Korean women of reproductive age and EOS in neonates in the last 20 years from a single center.

**Methods:**

From 2003 to 2022, microbiologic studies on genital/rectal swab pairs from females of reproductive age (15-49 years) were reviewed. Among the total number of tests, the number of GBS positive results were extracted and GBS detection rates per year were calculated. Multiple tests from the same person within a year were counted as once. Among the GBS isolates, the clindamycin resistance rate was calculated. The total study period was divided into two periods (period 1 for 2003-2012 and period 2 for 2013-2022) by 10 years and GBS detection rates and clindamycin resistance rates were compared (Chi-square test). EOS caused by GBS in neonates was investigated among all neonates who received sepsis work-up within 3 days after birth.

**Results:**

For the past 20 years, a total of 14,571 women were tested 16,879 times and GBS was isolated in 1,054 tests (6.2%). GBS detection rate increased from 1.7% (101/6,066) in period 1 to 8.8% (953/10,813) in period 2 (*p*< 0.001, OR 5.708, 95% CI 4.638-7.025). Among the GBS isolates, clindamycin resistance rates were 29.7% (30/101) in period 1 and 41.1% (392/953) in period 2 (*p*=0.026, OR 1.654, 95% CI 1.059-2.583).

In a separate database, among 9,280 neonates who underwent blood culture or CSF culture studies within 3 days after birth, 9 patients were diagnosed with EOS by GBS and 4 patients died within 30 days.

**Conclusion:**

Close monitoring is needed on the epidemiology of increasing GBS detection rate and clindamycin resistance rate in GBS isolates from Korean women of reproductive age.

**Disclosures:**

**All Authors**: No reported disclosures

